# Predefined-Time Super-Twisting Sliding Mode Control for Construction Robot with Arbitrary Initial Values

**DOI:** 10.3390/s26051654

**Published:** 2026-03-05

**Authors:** Hong-Bo Ai, Xin-Rong He, Chun-Wu Yin

**Affiliations:** 1 Design and Research Institute Co., Ltd., Xi’an University of Architecture and Technology, Xi’an 710055, China; aihongbo@xauat.edu.cn (H.-B.A.); hexinrong@xauat.edu.cn (X.-R.H.); 2 School of Information and Control Engineering, Xi’an University of Architecture and Technology, Xi’an 710055, China

**Keywords:** construction robot, fluid material, super-twisting sliding mode control, predefined time control, radial basis function

## Abstract

To tackle the practical engineering challenge that construction robots are required to track the reference trajectory completely and precisely, this study puts forward a control scheme based on the extended reference trajectory and develops a novel super-twisting sliding mode controller with predefined-time convergence capability. First, the influence mechanism of fluid materials on construction robots and their trajectory tracking control features are explored, and the design approach for the extended reference trajectory is elaborated. Subsequently, a nonsingular sliding surface with predefined-time convergence is constructed, and a RBF neural network with convergent weight vectors is established to approximate the composite disturbances existing in the robot system. On the basis of the proposed predefined-time convergent super-twisting control theory, a super-twisting sliding mode controller tailored for construction robots is devised, and the predefined-time convergence performance of the closed-loop system is theoretically validated. Numerical simulation results indicate that the proposed algorithm can guarantee that the construction robot’s angles move accurately along the actual reference trajectory, with the angular tracking error achieving a precision of 3 × 10^−6^ rad, thereby confirming the feasibility and effectiveness of the proposed method.

## 1. Introduction

In actual engineering scenarios, the output of a controlled system is generally required to strictly follow a preset trajectory. For instance, in the material spraying operation of construction robots, robotic arms equipped with various end-effectors must move along the preset trajectory to ensure the spraying quality of fluid materials; otherwise, it will lead to defects in the product quality. In practical applications, it is difficult to ensure that the initial state of the controlled system is completely consistent with that of the preset trajectory, and existing control strategies fail to enable the output of the controlled system to accurately track the preset reference trajectory (ReT). Therefore, researching the control strategy of construction robots to achieve complete and precise trajectory tracking (TraT) under arbitrary initial states has significant theoretical significance and broad prospects in engineering applications.

Regulating construction robots to perform building material spraying is essentially a process of adjusting the position of their end-effectors to follow a given trajectory. Thus, analyzing the characteristics of the dynamic model of construction robots according to their actual construction conditions and designing an adaptive controller with strong robustness and high tracking accuracy for them are the key strategies to improve technologies for the engineering application of construction robots. For the end-effector of construction robots to strictly follow a specified trajectory, the robot’s initial position must exactly match that of the ReT. Nevertheless, influenced by the operational environment, the initial position of construction robots is arbitrary in most cases, which makes it challenging for conventional control strategies and algorithms to achieve full and precise tracking of the ReT by the robot’s end-effector. To address this practical engineering problem, we propose a control strategy based on an extended ReT, which integrates a reaching phase before the original ReT. In this phase, robots with arbitrary initial positions converge towards the original ReT, while the original ReT is defined as the precise tracking phase, in which construction robots are guaranteed to strictly follow the trajectory. This approach effectively solves the problem of complete and accurate TraT control for construction robots with arbitrary initial states. To ensure the successful implementation of this control strategy, three key challenges must be resolved: first, the design of the extended trajectory for the reaching phase, which requires the extended ReT to have a continuous and concise form; second, the robot’s joint angles must converge to the extended ReT within a predefined time (PreDT), which requires the design of a predefined-time convergent controller for construction robots; third, the robotic arm is subjected to time-varying internal perturbations and external environmental disturbances during fluid delivery, so the designed controller must endow construction robots with strong robustness against uncertain factors such as internal and external parameter perturbations.

Considering that the end-effector path of construction robots does not need to pass through specific points and that any nonlinear function can be approximated by the Maclaurin series, this paper designs the ReT for the reaching phase using the Maclaurin series to address the extended trajectory design challenge. The second core issue of the extended ReT control strategy is essentially a time-optimal control problem. Early TraT control algorithms were mainly asymptotically stable controllers [1,2,3], for which the convergence time of TraT errors approaches infinity. To improve convergence speed, finite-time controllers were developed; however, the estimation of their upper bound (UperB) of convergence time is highly dependent on initial states and controller gains [4,5]. Subsequently, researchers proposed fixed-time control, where the UperB of the convergence time for TraT errors is solely related to controller gains [6,7]. Nevertheless, controller parameters govern the stability and dynamic performance of the controlled plant, and the convergence time of tracking errors remains unregulated. In recent years, a predefined-time convergent control approach with a presettable UperB of convergence time has been proposed and has become a key research direction [8,9]. Sanchez T. and co-workers introduced the concept of predefined-time stability and designed predefined-time sliding mode controllers (SMClers) for first-order uncertain systems [10] and second-order systems [11], respectively. Although these controllers feature presettable convergence time, their design is based on the stability criterion for fixed-time convergence. Ref. [12] established a stability criterion dedicated to predefined-time convergence and developed a practical predefined-time convergent controller for uncertain robotic manipulators. Ref. [13] further optimized the stability criterion for predefined-time convergence, enabling the controllers designed based on this revised criterion to have tunable gain parameters. However, the predefined-time convergent sliding mode surface (SMSur) in Ref. [13] includes fractional-order terms, which induce singularity and chattering issues in the controller. High-order SMC technology can effectively alleviate the chattering problem in traditional SMC, among which the super-twisting control algorithm is the most widely applied [14,15]. By embedding nonlinear transcendental functions and adopting a SMSur with super-twisting characteristics, the super-twisting SMC algorithm smooths discontinuous signals through integrators and significantly reduces chattering. Nevertheless, existing super-twisting SMC algorithms only ensure the finite-time convergence of TraT errors, and the precise analysis of their convergence time is quite complex.

The robotic arm of construction robots is equipped with fluid delivery pipelines, which leads to significant parameter perturbations of the arm; therefore, simple SMC cannot meet the robust control requirements of construction robots. To enhance the closed-loop system’s ability to resist external disturbances, technologies such as observers [16,17], neural networks [18,19], and fuzzy systems [20] must be adopted to estimate the uncertain components. Additionally, SMClers based on an estimation compensator should be constructed to improve robustness against uncertain disturbances. Neural networks are widely used to approximate the uncertain components in systems, but the update laws of neural network weights in existing neural network-based control algorithms lack convergence [21,22]. To address this issue, this paper designs an update law for the RBF weight vector with convergence characteristics to improve the convergence rate of the RBF weight vector.

Based on the above analysis, the contributions are summarized as:

(1) Innovation in control method: Existing research achievements in robot control lack the analysis of motion characteristics for construction robots. This paper analyzes the influence mechanism of fluid materials on spraying robots, and presents the tracking control characteristics of spraying robots according to the requirements of engineering applications. Aiming at the practical engineering problem that traditional control algorithms cannot guarantee, the complete and accurate tracking of the desired trajectory by the end-effector of spraying robots, this paper proposes a control strategy based on an extended desired trajectory, and provides the design method of the extended trajectory, which effectively solves the problem of applying control theory to practical engineering.
(2) Innovation in control theory: A novel predefined-time convergent super-twisting control method is constructed, and the corresponding design method of the super-twisting controller is presented, which enriches the theoretical studies on high-order SMC. Unlike traditional super-twisting control strategies that only guarantee the finite-time convergence of the SMSur, the super-twisting controller designed based on the proposed method ensures the predefined-time convergence of the SMSur, and the convergence time can be configured in accordance with engineering demands.
(3) Innovation in controller design: Aiming at the singularity and chattering problems of the predefined-time convergent SMSur proposed in Ref. [13], a predefined-time nonsingular sliding surface is proposed. The RBF with convergence characteristics is applied to approximate the composite disturbance of controlled system, and a predefined-time convergent super-twisting SMCler is designed for uncertain construction robots based on this sliding surface. This controller guarantees that the joint angle tracking errors for construction robots converge towards the ReT within a preset time and improves construction robots’ robustness against a variety of uncertain disturbance factors.

## 2. Dynamic Model and Characteristic Analysis of Construction Robots

In this section, the dynamic model of the construction robot is established. The influence mechanism of fluid materials moving through the manipulator and the conveying pipeline on the dynamic model of the robot is analyzed emphatically, and the particularity of the control of construction robots compared with traditional industrial robots is clarified. Finally, an extended trajectory control strategy is proposed to ensure that the construction robot can fully track the desired trajectory.

### 2.1. Model of Construction Robots

The dynamical system of construction robots is described as [4]

(1) $$D(\theta ,q(t))\ddot{q}+N(q(t),\dot{q}(t))\dot{q}+G(q(t))=u(t)+\bar{d}(t)$$

where $q(t),\dot{q}(t),\ddot{q}(t)$ denote the joint angle, angular velocity and angular acceleration of the construction robot; θ(t) represents the parameters of the robot; $D(\theta ,q),N(q,\dot{q}),G(q)$ stand for the inertia matrix, Coriolis and centrifugal force matrix, and gravity vector, with D(θ,q(t)) being an invertible matrix; and u(t) denotes the control input and $\bar{d}(t)$ represents the external disturbance.

### 2.2. Influence Mechanism of Fluid Materials on the Dynamic Model

When fluid materials are delivered through the robotic arm for spraying operations, the flow velocity of the fluid undergoes variations in three phases: start-up, steady flow and shutdown. Namely, the start-up phase $v_{f\max}$ where the flow velocity increases linearly from 0 to $v_{f\max}$, the steady flow phase $\left(t_{1}\leq t<t_{2}\right)$ where the flow velocity remains constant at $v_{f\max}$, and the shutdown phase $\left(t_{2}\leq t<t_{3}\right)$ where the flow velocity decreases linearly from $v_{f\max}$ to 0. The flow velocity in these three phases can be described as:

(2) $$v_{\mathrm{fluid}}(t)=\left\{\begin{array}{ll} \frac{v_{f\max}}{t_{1}}t & 0\leq t<t_{1}\\ v_{f\max} & t_{1}\leq t<t_{2}\\ \frac{v_{f\max}(t_{3}-t)}{t_{3}-t_{2}} & t_{2}\leq t<t_{3} \end{array}\right.$$

(1) Analysis of the Effects of Mass and Moment of Inertia

Although the fluid inside the pipeline is in flow, the mass of the fluid material retained in the pipeline remains constant, i.e.,

(3) $$m_{\mathrm{fluid}}(t)=\rho_{\mathrm{fluid}}A_{\mathrm{fluid}}L_{\mathrm{fluid}}$$

where $\rho_{\mathrm{fluid}}$ denotes the density of the fluid material, $A_{\mathrm{fluid}}$ the cross-sectional area of the pipeline, and $L_{\mathrm{fluid}}$ is the total length of the pipeline. When the length of the pipeline on the ith link is $L_{i,\mathrm{fluid}}$ and the vertical distance from the fluid centroid to the rotation axis of the ith joint is $r_{i}$, the mass $m_{i,\mathrm{fluid}}(t)$ and the moment of inertia $I_{i,\mathrm{fluid}}(t)$ of the fluid material on the ith link are given by

(4) $$\left\{\begin{array}{l} m_{i,\mathrm{fluid}}(t)=\frac{L_{i,\mathrm{fluid}}}{L_{\mathrm{fluid}}}m_{\mathrm{fluid}}(t)\\ I_{i,\mathrm{fluid}}(t)=m_{i,\mathrm{fluid}}(t){r_{i}}^{2} \end{array}\right.$$

It can be concluded from the above analysis that the fluid material induces variations in the mass and moment of inertia of the robotic arm. Denoting the nominal matrixes as $D_{0}(\theta ,q),N_{0}(q,\dot{q}),G_{0}(q)$ and the perturbation matrixes as $\Delta D(\theta ,q),\Delta N(q,\dot{q}),\Delta G(q)$, we obtain

(5) $$D(\theta ,q)=D_{0}(\theta ,q)+\Delta D(\theta ,q)$$

(6) $$N(q,\dot{q})=N_{0}(q,\dot{q})+\Delta N(q,\dot{q})$$

(7) $$G(q)=G_{0}(q)+\Delta G(q)$$

(2) Frictional Force

When fluid materials flow inside the attached pipelines, frictional force is generated between the fluid and the pipeline walls, which increases the resistance for the robotic arm to overcome friction and thus impairs the power output of the robotic arm. The frictional force between the fluid materials and the pipeline walls obeys Coulomb’s law of friction, and is given by

(8) $$F_{f}=(\mu_{0f}+k_{v}v_{\mathrm{fluid}}(t))m_{\mathrm{fluid}}g\cos\phi$$

where $\mu_{0f}$ is the initial friction coefficient, $k_{\upsilon }$ is the coefficient related to the flow velocity $v_{\mathrm{fluid}}(t)$, $m_{\mathrm{fluid}}$ is the mass of the fluid material in the pipeline, g denotes the gravitational acceleration, and ϕ is the angle between the pipeline and the horizontal direction. The equivalent friction torque is given by

(9) $$\tau_{f_{i}}(t)=r_{fi}F_{f}(t)$$

where $r_{fi}$ denotes the moment arm of the frictional force $F_{f}$ acting on the i-th joint, i.e., the vertical distance from the rotation center of the i-th joint to the line of action of the frictional force, which governs the magnitude of the frictional torque acting on the joint.

(3) Fluid Flow Pressure

To enable the fluid material to flow inside the pipeline and be delivered to the end-effector of the robotic arm, a certain pressure is required to drive the fluid flow. This pressure exerts a reaction force on the robotic arm, thereby disrupting the dynamic balance of the robotic arm. According to the Bernoulli equation in fluid mechanics, the force applied to the robotic arm via the pressure P of the fluid material inside the pipeline is given by

(10) $$F_{P}=\frac{8\mu_{P}L_{\mathrm{fluid}}Q}{r_{pp}^{2}}$$

where Q is the flow rate of the fluid material, $L_{\mathrm{fluid}}$ is the pipeline length, $r_{pp}$ is the pipeline radius, and $\mu_{P}$ is the dynamic viscosity of the fluid material. The equivalent pressure torque is given by

(11) $$\tau_{p_{i}}=r_{p_{i}}F_{p}$$

where $r_{pi}$ denotes the moment arm of the pressure $F_{P}$ acting on the i-th joint, through which the pressure can be converted into an equivalent driving torque on the joint.

Synthesizing the above influencing factors of fluid materials on the robot, let $x_{1}(t)=q(t),x_{2}(t)=\dot{q}(t)$ denote the resultant effect, and the dynamic equation is given by

(12) $$\left\{\begin{array}{l} {\dot{x}}_{1}(t)=x_{2}(t)\\ {\dot{x}}_{2}(t)=D_{0}^{-1}(\theta ,x_{1})(-N_{0}(x_{1},x_{2})x_{2}(t)-G_{0}(x_{1}))+D_{0}^{-1}(\theta ,x_{1})u(t)+d(t) \end{array}\right.$$

where $d(t)=D_{0}^{-1}(t)[-\Delta D(\theta ,x_{1}){\dot{x}}_{2}(t)-\Delta N(x_{1},x_{2})x_{2}(t)-\Delta G(x_{1})+\bar{d}(t)+\tau_{f}(t)+\tau_{p}(t)]$ is a composite disturbance term with strong time varying characteristics.

### 2.3. Analysis of Trajectory Tracking Control Characteristics for Construction Robots

Material smearing or spraying operations performed by construction robots essentially boil down to the precise trajectory tracking control of the robotic arm’s end-effector position. Considering the actual engineering application environment of construction robots, the following control challenges must be addressed when designing trajectory tracking (TraT) control algorithms for such robots:

(1) High-precision full tracking: The end-effector position of the robot must strictly and accurately follow the specified trajectory; otherwise, product quality defects will occur.
(2) Arbitrary initial state problem: Spraying operations are repetitive tasks that require the robotic arm’s end-effector to be repositioned in each working cycle. Affected by factors such as robotic arm vibration and fluid material splashing, the repositioning of the end-effector inevitably exhibits randomness, making it difficult to guarantee that the end-effector can be accurately placed at the predetermined position.

### 2.4. Extended ReT Strategy

**Figure 1:**
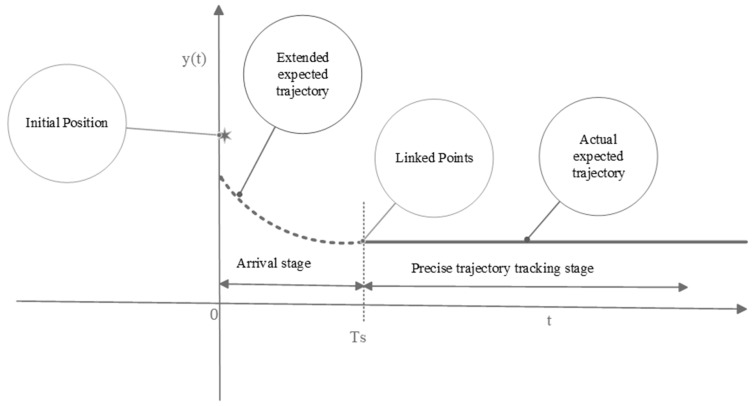
Expected trajectory expansion strategy.

When the initial state of the construction robot cannot be strictly aligned with the starting point of the ReT, conventional control methods can only guarantee TraT within a local range, making it difficult to achieve global precise tracking. To meet the stringent requirements for fully precise TraT in practical engineering applications, this paper proposes a control strategy based on an extended ReT, as illustrated in Figure 1.

The extended ReT is divided into two parts: the first is an additionally added reaching phase, which is designed to address the convergence of the robot’s joint angle q(t) to the extended ReT ${\bar{x}}_{r}(t)$ with arbitrary initial values. The second phase is dedicated to precise TraT; that is, the stage where the joint angles of the robotic arm are required to realize full and accurate tracking of the ReT ${\bar{x}}_{r}(t)$ in actual engineering scenarios. The extended ReT is not restricted to any specific expression form, and since any function can be represented by a Maclaurin series expansion, a quadratic power function is selected as the extended ReT. The extended ReT is given by

(13) $${\bar{x}}_{r}(t)=\left\{\begin{array}{l} At^{2}+Bt+C\\ x_{r}(t-T_{s}) \end{array}\right.\begin{array}{c} 0\leq t<T_{s}\\ T_{s}\leq t\leq T \end{array}$$

where parameter C determines the initial position of the extended trajectory; $A=\frac{{\dot{x}}_{r}(0)T_{s}-x_{r}(0)+C}{T_{s}^{2}},\;B=\frac{2x_{r}(0)-{\dot{x}}_{r}(0)T_{s}-2C}{T_{s}}$. $T_{s}$ denotes the time it takes for the robotic arm’s end-effector to converge to the original ReT $x_{r}(t)$, which can be predefined in accordance with practical engineering application requirements; $x_{r}(t-T_{s})$ denotes the time delay of $T_{s}$ imposed on the original ReT $x_{r}(t)$.

The extended trajectory method can effectively address the challenge of high-precision TraT for the robot’s end-effector under arbitrary initial states, yet its core challenge lies in ensuring that the end-effector of the manipular accurately tracks the extended ReT ${\bar{x}}_{r}(t)$ within the specified time $T_{s}$.

Define the TraT error as:

(14) $$e(t)=x_{1}(t)-{\bar{x}}_{r}(t)$$

## 3. Predefined-Time Convergent Super-Twisting Control Theorem

This section presents the structure of the traditional super-twisting controller and analyzes its limitations. On this basis, a super-twisting sliding mode control method with predefined-time convergence is proposed, and the corresponding theoretical proof is completed.

### 3.1. Finite-Time Convergent Super-Twisting Algorithm

For the simple second-order system:

(15) $$\left\{\begin{array}{l} {\dot{z}}_{1}(t)=z_{2}(t)\\ {\dot{z}}_{2}(t)=f(z(t),t)+b(t)u(t) \end{array}\right.,z(0)=z_{0}$$

where$z={\left[z_{1},z_{2}\right]}^{Τ}$ is the state, f(z(t),t),b(t) are continuous functions, b(t) is invertible, and u(t) is the input signal.

If the SMSur s(t) satisfies Equation (16), then s(t) is finite-time stable [15].

(16) $$\dot{s}(t)=-k_{1}s^{\left[1/2\right]}(t)+v(t)$$

(17) $$\dot{v}(t)=-k_{2}\mathrm{sign}(s(t))$$

where $k_{1}>0,k_{2}>0$ are the gains to be designed, and $s^{\left[1/2\right]}(t)=|s(t){|}^{1/2}\mathrm{sign}(s(t))$, and sign(·) is the sign function. Function v(t) is an intermediate variable.

Unlike traditional SMC, the super-twisting variant generates the actual control input through integration and eliminates the high-frequency switching term, thereby suppressing chattering. Furthermore, the associated control algorithm ensures the finite-time stability of the sliding surface.

For the nonlinear system (15), a linear SMSur is designed as $s(t)=cz_{1}(t)+z_{2}(t)$, and the corresponding super-twisting SMCler is given by

(18) $$u(t)=b^{-1}(t)(-f(z(t),t)-cz_{2}(t)-k_{1}s^{\left[1/2\right]}(t)+v(t))$$

(19) $$\dot{v}(t)=-k_{2}\mathrm{sign}(s(t))$$

**Remark 1.**  *The convergence process of SMC comprises two stages: the reaching phase, during which state variables approach the SMSur, and the sliding phase, where they subsequently move toward the origin. However, conventional super-twisting algorithms merely guarantee finite-time convergence to the SMSur, without ensuring the subsequent motion toward the equilibrium point. Meanwhile, the SMSur has the property of asymptotic stability, such that the state variables on the SMSur converge to zero asymptotically. Therefore, the super-twisting SMCler (18) cannot guarantee that the state variables of the system converge to the origin within a prespecified time, which fails to meet the engineering requirement of construction robots for convergence within a prespecified time*.

### 3.2. Predefined-Time Convergent Super-Twisting Control Theory

**Lemma 1 [22].**  *For the given nonlinear system *$\dot{z}(t)=f(z(t),t),z(0)=z_{0}$*, if there exists a positive definite function*V(z)*that satisfies the following inequality:*

(20) $$\dot{V}(z)\leq -\frac{\pi }{pT_{c}\sqrt{\alpha \beta }}\left(\alpha V^{1-\frac{p}{2}}(z)+\beta V^{1+\frac{p}{2}}(z)\right)$$

*then the system *$\dot{z}(t)=f(z(t),t),z(0)=z_{0}$* is PreDT stable, where *$T_{c}>0$* denotes the preset convergence time, and the parameters *α* and *β*are positive constants*, 0<p≤2.

**Corollary 1.**  * For the given system *$\dot{z}(t)=f(z(t),t),z(0)=z_{0}$, *when *p=1, *the stability criterion for PreDT conergence is: *

(21) $$\dot{V}(z)\leq -\frac{\pi }{T_{c}\sqrt{\alpha \beta }}\left(\alpha V^{1/2}(z)+\beta V^{3/2}(z)\right)$$

A novel super-twisting control theory with PreDT convergence is presented as follows.

**Theorem 1.**  *If the sliding surface *s(t)* satisfies Equation (22), then the sliding surface *s(t) *is PreDT stable.*

(22) $$\dot{s}(t)=\frac{\pi \left(-k_{c1}s^{\left[1/2\right]}(t)-k_{c2}s^{\left[3/2\right]}(t)+v(t)+v^{3}(t)\right)}{T_{cp}\sqrt{\alpha \beta }}$$

(23) $$\dot{v}(t)=-\frac{\pi }{T_{cp}\sqrt{\alpha \beta }}(k_{c3}+|s|)\mathrm{sign}(s)$$

*where *$k_{c1}>0,k_{c2}>0$* are the gains to be designed*, $T_{cp}$* is the preset convergence time, and *α,β* are determined by *$k_{c1},k_{c2}$. v(t)* is an intermediate variable.*

**Proof.**  Construct Lyapunov function $V_{1}(s(t))=0.5\xi^{Τ}(t)P\xi (t)$, where $\xi (t)=\left[\begin{array}{c} s^{\left[1/2\right]}(t)\\ v(t) \end{array}\right],P=\left[\begin{array}{cc} 2(k_{c3}+k_{c1}^{2}) & -2k_{c1}\\ -2k_{c1} & 4 \end{array}\right]$. Then

(24) $$\lambda_{\min}\{P\}||\xi |{|}_{2}^{2}\leq V_{1}(s)\leq \lambda_{\max}\{P\}||\xi |{|}_{2}^{2}\Rightarrow ||\xi |{|}_{2}^{2}\geq \frac{V_{1}(s)}{\lambda_{\max}\{P\}}\Rightarrow ||\xi |{|}_{2}\geq \frac{{V_{1}}^{1/2}(s)}{\lambda_{\max}^{1/2}\{P\}}$$

where $||\xi |{|}_{2}^{2}=|s|+v^{2}$, $\lambda_{\min}\{P\}$/$\lambda_{\max}\{P\}$ represents the minimum//maximum eigenvalue of matrix P and $||\xi |{|}_{2}$ represents the 2-norm of vector ξ. Then $||\xi |{|}_{2}^{2}\geq |s|\Leftrightarrow |s{|}^{1/2}|\leq ||\xi |{|}_{2}$ is hold and

(25) $$\begin{array}{ll} \frac{ds^{\left[1/2\right]}(t)}{dt} & =\frac{1}{2}|s(t){|}^{-1/2}\dot{s}(t)\\ & =\frac{\pi }{T_{cp}\sqrt{\alpha \beta }}\frac{\left(-k_{c1}s^{\left[1/2\right]}(t)-k_{c2}s^{\left[3/2\right]}(t)+v(t)+v^{3}(t)\right)}{2|s(t){|}^{1/2}} \end{array}$$

Thus, we have

(26) $$\begin{array}{ll} \dot{\xi }(t) & =\left[\begin{array}{c} \frac{\pi }{T_{cp}\sqrt{\alpha \beta }}\frac{-k_{c1}s^{\left[1/2\right]}(t)-k_{c2}s^{\left[3/2\right]}(t)+v(t)+v^{3}(t)}{2|s(t){|}^{1/2}}\\ -\frac{\pi }{T_{cp}\sqrt{\alpha \beta }}(k_{c3}+|s(t)|)\mathrm{sign}(s(t)) \end{array}\right]\\ & ≜-\frac{\pi }{T_{cp}\sqrt{\alpha \beta }}\frac{1}{2|s(t){|}^{1/2}}\left(Q_{1}\xi (t)+Q_{2}\xi^{3}(t)\right) \end{array}$$

where $Q_{1}=\left[\begin{array}{cc} k_{c1} & -1\\ 2k_{c3} & 0 \end{array}\right],Q_{2}=\left[\begin{array}{cc} k_{c2} & -1\\ 2 & 0 \end{array}\right]$, then denote

(27) $$H_{1}=PQ_{1}=\left[\begin{array}{cc} 2k_{c1}^{3}-2k_{c1}k_{c3} & -2(k_{c3}+k_{c1}^{2})\\ 8k_{c3}-2k_{c1}^{2} & 2k_{c1} \end{array}\right],H_{2}=PQ_{2}=\left[\begin{array}{cc} 2k_{c1}^{2}k_{c2}+2k_{c2}k_{c3}-4k_{c1} & -2(k_{c3}+k_{c1}^{2})\\ -2k_{c1}k_{c2}+8 & 2k_{c1} \end{array}\right]$$

when $k_{c1}>\sqrt{k_{c3}}$ holds, $H_{1}$ and $H_{2}$ are constant matrices, and then

(28) $$\lambda_{\min}(H_{1})||\xi |{|}_{2}^{2}\leq \xi^{Τ}H_{1}\xi \leq \lambda_{\max}(H_{1})||\xi |{|}_{2}^{2},\;\;\;\;\lambda_{\min}(H_{2})||\xi |{|}_{2}^{2}\leq \xi^{Τ}H_{2}\xi \leq \lambda_{\max}(H_{2})||\xi |{|}_{2}^{2}$$

Then

(29) $$\begin{array}{ll} \dot{V}(s) & =-\frac{\pi }{T_{cp}\sqrt{\alpha \beta }}\frac{\xi^{Τ}(t)P\left(Q_{1}\xi (t)+Q_{2}\xi^{3}(t)\right)}{2|s(t){|}^{1/2}}\\ & =-\frac{\pi }{T_{cp}\sqrt{\alpha \beta }}\frac{\left(\xi^{Τ}(t)H_{1}\xi (t)+\xi^{Τ}(t)H_{2}\xi^{3}(t)\right)}{2|s(t){|}^{1/2}}\\ & \leq -\frac{\pi }{T_{cp}\sqrt{\alpha \beta }}\frac{\lambda_{\max}(H_{1})||\xi (t)|{|}_{2}^{2}+\lambda_{\max}(H_{2})||\xi (t)|{|}_{2}^{3}}{2|s(t){|}^{1/2}} \end{array}$$

Because $|s{|}^{1/2}|\leq ||\xi |{|}_{2}$, so $\frac{1}{|s{|}^{1/2}}\geq \frac{1}{||\xi |{|}_{2}}\Rightarrow \frac{-1}{|s{|}^{1/2}}\leq -\frac{1}{||\xi |{|}_{2}}$, and $||\xi |{|}_{2}\geq \frac{V^{1/2}(s)}{\lambda_{\max}^{1/2}\{P\}}\Rightarrow -||\xi |{|}_{2}\leq$ $-\frac{V^{1/2}(s)}{\lambda_{\max}^{1/2}\{P\}}$, then

(30) $$\begin{array}{ll} {\dot{V}}_{1}(s(t))\leq & \frac{\pi \left(\lambda_{\max}(H_{1})||\xi (t)|{|}_{2}^{2}+\lambda_{\max}(H_{2})||\xi |{|}_{2}^{4}\right)}{-T_{cp}\sqrt{\alpha \beta }||\xi (t)|{|}_{2}}\\ & \leq -\frac{\pi }{T_{cp}\sqrt{\alpha \beta }}\left(\alpha {V_{1}}^{1/2}(s(t))+\beta V_{1}^{3/2}(s(t))\right) \end{array}$$

where $\alpha =\frac{\lambda_{\max}(H_{1})}{\lambda_{\max}^{1/2}\{P\}},\beta =\frac{\lambda_{\max}(H_{2})}{\lambda_{\max}^{1/2}\{P\}}$. According to Lemma 1, ξ(t) achieves PreDT convergence. Moreover, since $\xi (t)=\left[\begin{array}{c} s^{\left[1/2\right]}(t)\\ v(t) \end{array}\right]$ holds, SMSur s(t) is PreDT convergent. □

## 4. Super-Twisting Controller Design

**Figure 2:**
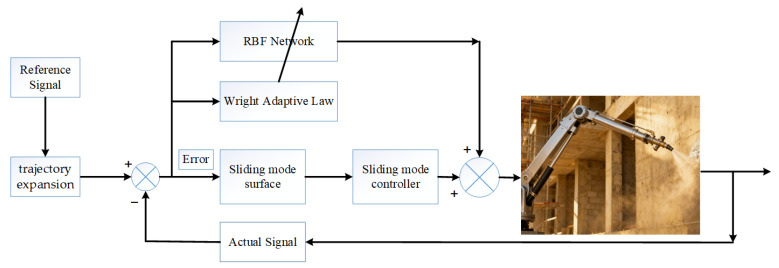
System architecture diagram.

In this section, a sliding surface with predefined-time convergence is first constructed. On this basis, a corresponding super-twisting sliding mode controller is designed for the construction robot. Finally, the predefined-time convergence of the proposed controller is rigorously proved theoretically. The system architecture diagram of the construction robot is shown in Figure 2.

### 4.1. Controller Design

Step 1: Construct a PreDT convergent nonsingular sliding surface.

To guarantee the controllability of the convergence time’s UperB for the tracking error, we develop a nonsingular SMSur endowed with the characteristic of PreDT convergence.

(31) $$s_{NS}(t)=\dot{e}(t)+\frac{\pi \left(k_{s1}\varphi (t)+k_{s2}e^{\left[3/2\right]}(t)\right)}{T_{sc}\sqrt{k_{s1}k_{s2}}}$$

where $k_{s1},k_{s2}>0$, $0<\delta_{e}<1$, $l_{2},l_{3}>0$, $l_{1}=\delta_{e}^{\frac{-1}{2}}-l_{2}\delta_{e}-l_{3}\delta_{e}^{2}$, $T_{sc}$ is the preset convergence time of the SMSur. The components of $\varphi ={\left[\varphi_{1},\varphi_{2},\ldots ,\varphi_{n}\right]}^{Τ}$ are

(32) $$\varphi_{i}(t)=\varphi (e_{i}(t))=\left\{\begin{array}{l} e_{i}^{\left[1/2\right]}(t),\;\;\;\;\;\;\;\;\;\;\;\;\;\;\;\;\;\;\;\;\;\;\;\;|e_{i}(t)|>\delta_{e}\\ l_{1}e_{i}(t)+l_{2}e_{i}^{\left[2\right]}(t)+l_{3}e_{i}^{\left[3\right]}(t),\;|e_{i}(t)|\leq \delta_{e} \end{array}\right.$$

**Remark 2.**  *If* $e_{i}>0$* holds, the right limit of *$\varphi (e_{i}(t))$* at *$\delta_{e}$* is *$\underset{e_{i}\to \delta_{e}^{+}}{\lim}\varphi (e_{i}(t))=\underset{e_{i}\to \delta_{e}^{+}}{\lim}e_{i}^{\left[1/2\right]}=\underset{e_{i}\to \delta_{e}^{+}}{\lim}|e_{i}{|}^{1/2}\mathrm{sign}(e_{i})=\underset{e_{i}\to \delta_{e}^{+}}{\lim}|e_{i}{|}^{1/2}=\delta_{e}^{1/2}$*; the left limit of *$\varphi (e_{i}(t))$* at *$\delta_{e}$* is *$\underset{e_{i}\to \delta_{e}^{-}}{\lim}\varphi (e_{i}(t))=\underset{e_{i}\to \delta_{e}^{-}}{\lim}(l_{1}e_{i}+l_{2}e_{i}^{\left[2\right]}+l_{3}e_{i}^{\left[3\right]})=\underset{e_{i}\to \delta_{e}^{-}}{\lim}(l_{1}e_{i}+l_{2}|e_{i}{|}^{2}\mathrm{sign}(e_{i})$$+l_{3}|e_{i}{|}^{3}\mathrm{sign}(e_{i}))=l_{1}\delta_{e}+l_{2}\delta_{e}^{2}+l_{3}e_{e}^{3}=\delta_{e}^{1/2}$*. It can be seen that the left and right limits of *$\varphi (e_{i}(t))$* at *$\delta_{e}$* are equal, so *$\varphi (e_{i}(t))$* is continuous at *$\delta_{e}$*. Similarly, it can be proven that *$\varphi (e_{i}(t))$* is continuous at *$\delta_{e}$*. In summary, the function *$\varphi (e_{i}(t))$* is continuous at all piecewise points, which can efficiently eliminate the abrupt variation in the control input arising from the discontinuity of the piecewise function.*

Differentiating the SMSur $s_{NS}(t)$, we obtain

(33) $${\dot{s}}_{NS}(t)=D_{0}^{-1}(-N_{0}x_{2}-G_{0}+u)+d-{\ddot{x}}_{d}+\frac{\pi \left(k_{s1}\dot{\varphi }(t)+\frac{3}{2}k_{s2}e^{\left[1/2\right]}(t)\dot{e}(t)\right)}{T_{sc}\sqrt{k_{s1}k_{s2}}}$$

Step 2: Approximation of Uncertain Systems Based on RBF Neural Network

For any continuous function, there exists a neural network that can approximate the function with arbitrary precision. Considering the strong time-varying characteristics of the uncertain part d(t), this paper adopts the RBF to approximate the system model and external disturbance d(t), i.e.,

(34) d(t)=W(t)Φ(X(t))

where $\Phi (X(t))={\left[\Phi_{1}(X),\Phi_{2}(X),\cdots ,\Phi_{n}(X)\right]}^{Τ}$ and n>1 denote the number of nodes of the neural network, W(t) is the optimal weight matrix, $X(t)=(e(t),\dot{e}(t))$ represents the input vector of the neural network, and the radial basis function $\Phi_{i}(X(t))$ is given by [21]

(35) $$\Phi_{i}(X(t))=\exp(-\frac{\left||X(t)-a_{i}|\right|}{{b_{i}}^{2}})$$

where $a_{i}$ and $b_{i}$ are the center vector and width, and their values can be randomly assigned in practical engineering. In practice, it is challenging to derive the optimal weight matrix W(t), and only its estimated value $\hat{W}(t)$ can be attained, i.e.,

(36) $$d(t)=\hat{W}(t)\Phi (X(t))+\Lambda (X(t))$$

where the approximation error Λ(X) satisfies $||\Lambda (X)||\leq \Lambda^{*}$, and $\Lambda^{*}>0$ is the precision index. When $\Lambda^{*}$ is sufficiently small, Λ(X) is negligible, and thus d(t) is approximated by $\hat{W}(t)\Phi (X(t))$, i.e.,

(37) $$\hat{d}(t)=\hat{W}(t)\Phi (X(t))$$

where the update law of $\hat{W}(t)$ is

(38) $$\dot{\hat{W}}(t)={\left[\begin{array}{cc} \xi_{1,NS}^{Τ}(t)P\chi_{1}(t) & \xi_{2,NS}^{Τ}(t)P\chi_{2}(t) \end{array}\right]}^{Τ}\Phi^{Τ}(X(t))$$

where $\xi_{i,NS}(t)={\left[\begin{array}{cc} s_{i,NS}^{\left[1/2\right]}(t) & v_{i}(t) \end{array}\right]}^{Τ}$,$\chi_{i}(t)={\left[\begin{array}{cc} \frac{1}{2|s_{i,NS}(t){|}^{1/2}} & 0 \end{array}\right]}^{Τ}$.

Step 3: Controller

A PreDT convergent super-twisting controller is designed as follows:

(39) $$u(t)=D_{0}\left(\begin{array}{l} \frac{\pi (k_{s1}\dot{\varphi }(t)+1.5k_{s2}e^{\left[1/2\right]}(t)\dot{e}(t))}{-T_{sc}\sqrt{k_{s1}k_{s2}}}-\hat{W}(t)\Phi (X)\\ +{\ddot{x}}_{d}(t)+\frac{\pi (-k_{c1}s_{NS}^{\left[1/2\right]}(t)-k_{c2}s_{NS}^{\left[3/2\right]}(t)+v(t)+v^{3}(t))}{T_{cp}\sqrt{\alpha \beta }} \end{array}\right)+N_{0}x_{2}(t)+G_{0}$$

(40) $$\dot{v}(t)=-\frac{\pi }{T_{cp}\sqrt{\alpha \beta }}(k_{c3}+|s_{NS}(t)|)\mathrm{sign}(s_{NS}(t))$$

In Equation (39), $\frac{\pi (k_{s1}\dot{\varphi }(t)+1.5k_{s2}e^{\left[1/2\right]}(t)\dot{e}(t))}{-T_{sc}\sqrt{k_{s1}k_{s2}}}+{\ddot{x}}_{d}(t)$ represents the equivalent controller of the sliding mode controller, which is used to offset the influences of the sliding surface and the desired trajectory on the system. The neural network approximation $\hat{W}(t)\Phi (X)$ is adopted to compensate for the compound disturbances of the system. $\frac{\pi (-k_{c1}s_{NS}^{\left[1/2\right]}(t)-k_{c2}s_{NS}^{\left[3/2\right]}(t)+v(t)+v^{3}(t))}{T_{cp}\sqrt{\alpha \beta }}$ denotes the reaching law of the super-twisting sliding mode controller, which guarantees the predefined-time convergence of the closed-loop system.

### 4.2. Stability Proof

**Theorem 2.**  *For the construction robot (12), when the controller is the PreDT super-twisting controller (39), the SMSur (31) achieves PreDT convergence. Moreover, the tracking error within the SMSur is PreDT convergent, and thus the TraT error of the construction robot is also PreDT convergent.*

**Proof.**  Substituting the controller (39) into (33) yields:

(41) $${\dot{s}}_{NS}(t)=\tilde{d}(t)+\frac{\pi (-k_{c1}s_{NS}^{\left[1/2\right]}(t)-k_{c2}s_{NS}^{\left[3/2\right]}(t)+v(t)+v^{3}(t))}{T_{cp}\sqrt{\alpha \beta }}$$

then

(42) $$\frac{ds_{i,NS}^{\left[1/2\right]}(t)}{dt}=\frac{1}{2}|s_{i,NS}(t){|}^{-1/2}\;{\dot{s}}_{i,NS}(t)=\frac{{\tilde{d}}_{i}(t)}{2|s_{i,NS}(t){|}^{1/2}}+\frac{\pi \left(-k_{c1}s_{i,NS}^{\left[1/2\right]}(t)-k_{c2}s_{i,NS}^{\left[3/2\right]}(t)+v_{i}(t)+v_{i}^{3}(t)\right)}{2T_{cp}\sqrt{\alpha \beta }|s_{i,NS}{|}^{1/2}}$$

(43) $${\dot{\xi }}_{i,NS}=\left[\begin{array}{c} \frac{\pi }{T_{cp}\sqrt{\alpha \beta }}\frac{\left(-k_{c1}s_{i,NS}^{\left[1/2\right]}-k_{c2}s_{i,NS}^{\left[3/2\right]}+v_{i}+v_{i}^{3}\right)}{2|s_{i,NS}{|}^{1/2}}\\ -\frac{\pi }{T_{cp}\sqrt{\alpha \beta }}(k_{c3}+|s_{i,NS}|)\mathrm{sign}(s_{i,NS}) \end{array}\right]+\chi_{i}{\tilde{d}}_{i}$$

Define the Lyapunov function as

(44) $$V_{2}(s_{NS})=\sum_{i=1}^{2}\frac{1}{2}\xi_{i,NS}^{Τ}(t)P_{i}\xi_{i,NS}(t)+\frac{1}{2}\mathrm{tr}(\tilde{W}{\left(t\right)}^{Τ}\tilde{W}(t))$$

Differentiating $V_{2}(s_{NS})$ yields

(45) $$\begin{array}{ll} {\dot{V}}_{2}(s_{NS})= & -\mathrm{tr}(\tilde{W}{\left(t\right)}^{Τ}\dot{\hat{W}}(t))+\sum_{i=1}^{2}\xi_{i,NS}^{Τ}P\left(\begin{array}{l} \left[\begin{array}{l} \frac{\pi }{T_{cp}\sqrt{\alpha \beta }}\frac{\left(-k_{c1}s_{i,NS}^{\left[1/2\right]}-k_{c2}s_{i,NS}^{\left[3/2\right]}+v_{i}+v_{i}^{3}\right)}{2|s_{i,NS}(t){|}^{1/2}}\\ -\frac{\pi }{T_{cp}\sqrt{\alpha \beta }}(k_{c3}+|s_{i,NS}(t)|)\mathrm{sign}(s_{i,NS}(t)) \end{array}\right]\\ +\chi_{i}(t){\tilde{d}}_{i}(t) \end{array}\right)\\ & \leq \frac{\pi \left(\alpha {V_{1}}^{1/2}(s_{1,NS})+\beta V_{1}^{3/2}(s_{1,NS})\right)}{-T_{cp}\sqrt{\alpha \beta }}-\frac{\pi \left(\alpha V_{1}^{1/2}(s_{2,NS})+\beta V_{1}^{3/2}(s_{2,NS})\right)}{T_{cp}\sqrt{\alpha \beta }}+\sum_{i=1}^{2}\xi_{i,NS}^{Τ}P\chi_{i}{\tilde{d}}_{i}-\mathrm{tr}({\tilde{W}}^{Τ}\dot{\hat{W}})\\ & =\frac{\pi \left(\alpha {V_{1}}^{1/2}(s_{1,NS})+\beta {V_{1}}^{3/2}(s_{1,NS})\right)}{-T_{cp}\sqrt{\alpha \beta }}-\frac{\pi \left(\alpha {V_{1}}^{1/2}(s_{2,NS})+\beta {V_{1}}^{3/2}(s_{2,NS})\right)}{T_{cp}\sqrt{\alpha \beta }}\\ & +\left[\begin{array}{cc} \xi_{1,NS}^{Τ}P\chi_{1} & \xi_{2,NS}^{Τ}P\chi_{2} \end{array}\right]\tilde{W}\Phi (X)-\mathrm{tr}({\tilde{W}}^{Τ}\dot{\hat{W}})\\ & =\frac{\pi \left(\alpha {V_{1}}^{1/2}(s_{1,NS})+\beta {V_{1}}^{3/2}(s_{1,NS})\right)}{-T_{cp}\sqrt{\alpha \beta }}-\frac{\pi \left(\alpha {V_{1}}^{1/2}(s_{2,NS})+\beta {V_{1}}^{3/2}(s_{2,NS})\right)}{T_{cp}\sqrt{\alpha \beta }}\\ & +\mathrm{tr}\left\{{\tilde{W}}^{Τ}\left({\left[\begin{array}{cc} \xi_{1,NS}^{Τ}P\chi_{1} & \xi_{2,NS}^{Τ}P\chi_{2} \end{array}\right]}^{Τ}\Phi^{Τ}-\dot{\hat{W}}\right)\right\}\\ & \leq -\frac{\pi }{T_{cp}\sqrt{\alpha 2^{-1/2}\beta }}\left(\alpha {V_{2}}^{1/2}(s_{NS})+2^{-1/2}\beta {V_{2}}^{3/2}(s_{NS})\right) \end{array}$$

According to Corollary 1, the SMSur $s_{NS}(t)$ converges to zero in PreDT. When $s_{NS}(t)=0$ holds, then

(46) $$\dot{e}(t)=\frac{\pi \left(k_{s1}\varphi (t)+k_{s2}e^{\left[3/2\right]}(t)\right)}{-T_{sc}\sqrt{k_{s1}k_{s2}}}$$

Construct a Lyapunov function $V_{e}(t)=0.5e{\left(t\right)}^{Τ}e(t)$, then

(47) $${\dot{V}}_{e}(t)=-e{\left(t\right)}^{Τ}\left(\frac{\pi \left(k_{s1}\varphi (t)+k_{s2}e^{\left[3/2\right]}(t)\right)}{T_{sc}\sqrt{k_{s1}k_{s2}}}\right)$$

(1) If $|e_{i}|>\delta_{e}$, then $\varphi (t)=e^{\left[1/2\right]}(t)$, so
  (48) $$\begin{array}{ll} {\dot{V}}_{e}(t) & =-e{\left(t\right)}^{Τ}\left(\frac{\pi \left(k_{s1}e^{\left[1/2\right]}(t)+k_{s2}e^{\left[3/2\right]}(t)\right)}{T_{sc}\sqrt{k_{s1}k_{s2}}}\right)\\ & =\frac{\pi (k_{s1}e{\left(t\right)}^{Τ}e^{\left[1/2\right]}(t)+k_{s2}e{\left(t\right)}^{Τ}e^{\left[3/2\right]}(t))}{-T_{sc}\sqrt{k_{s1}k_{s2}}}\\ & =-\frac{2\pi }{T_{sc}\sqrt{{\bar{k}}_{s1}{\bar{k}}_{s2}}}({\bar{k}}_{s1}V_{e}^{3/4}(t)+{\bar{k}}_{s2}V_{e}^{5/4}(t)) \end{array}$$
  where ${\bar{k}}_{s1}=2^{3/4}k_{s1},{\bar{k}}_{s2}=2^{5/4}k_{s2}$. Based on Lemma 1, the tracking error e(t) converges within the PreDT $T_{sc}$.
(2) if $|e_{i}|\leq \delta_{e}$, then $\varphi (t)=l_{1}e(t)+l_{2}e^{\left[2\right]}(t)+l_{3}e^{\left[3\right]}(t)$, so

(49) $$\begin{array}{l} {\dot{V}}_{e}(t)=\frac{\pi e{\left(t\right)}^{Τ}\left(k_{s1}(l_{1}e(t)+l_{2}e^{\left[2\right]}(t)+l_{3}e^{\left[3\right]}(t))+k_{s2}e^{\left[3/2\right]}(t)\right)}{-T_{sc}\sqrt{k_{s1}k_{s2}}}\\ =\frac{\pi }{-T_{sc}\sqrt{k_{s1}k_{s2}}}\left(2k_{s1}l_{1}V_{e}(t)+2^{\frac{3}{2}}k_{s1}l_{2}{V_{e}}^{\frac{3}{2}}(t)+2^{2}k_{s1}l_{3}{V_{e}}^{2}(t)+2^{5/4}k_{s2}{V_{1}}^{1/2}(t)\right)\\ \leq 0 \end{array}$$

Based on the Lyapunov stability theory, the tracking error e(t) will converge to zero exponentially. Since the value of $\delta_{e}$ is small, when the tracking error satisfies $|e_{i}|\leq \delta_{e}$, it follows from Equation (49) that the tracking error will also converge to zero rapidly. □

## 5. Numerical Simulation Verification

In this section, the parameter settings for numerical simulations of the construction robot are first established. Comparative simulations are then conducted from three perspectives, initial conditions, controller types, and the inclusion of neural network compensation, so as to verify the superiority of the proposed algorithm.

### 5.1. Simulation Object and Parameter Setting

The relevant parameters of the 2-DOF construction robot are listed in Table 1.

### 5.2. Analysis of Trajectory Tracking Performance Under Different Initial Values

**Figure 3:**
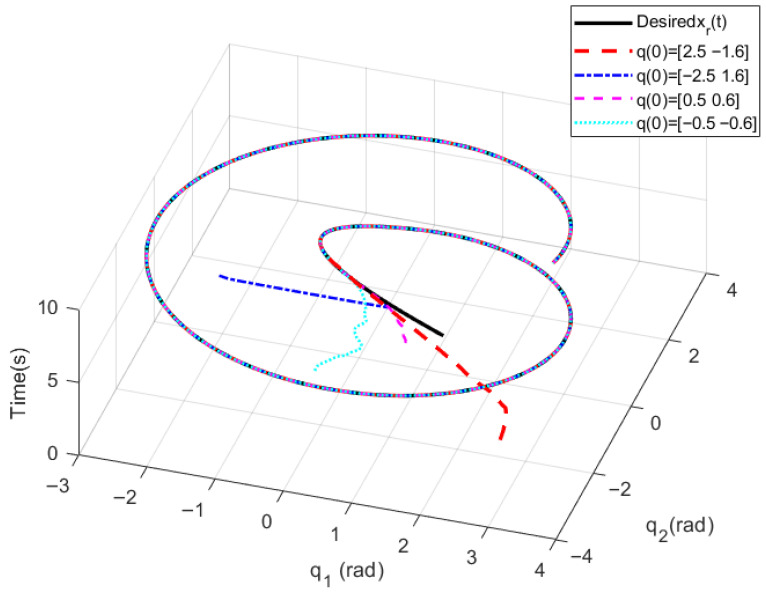
Three-dimensional diagram of angle tracking.

**Figure 4:**
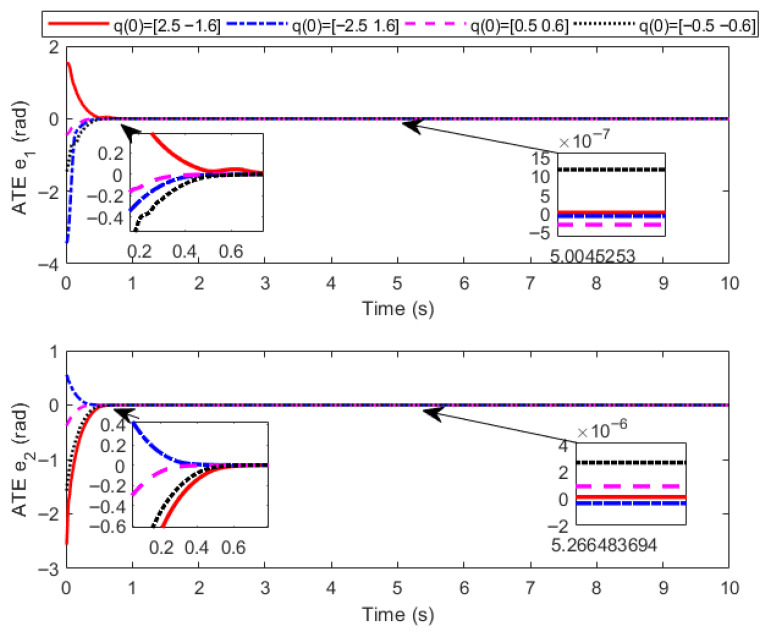
Diagram of angle tracking error.

**Figure 5:**
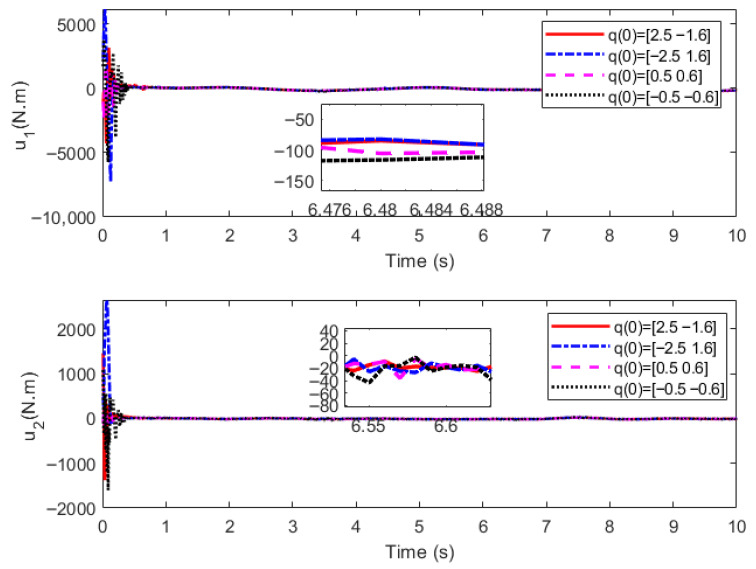
Control input.

Initial angles q(0)=[2.5,−1.6], q(0)=[−2.5,1.6], q(0)=[0.5,0.6], q(0)=[−0.5,−0.6] and initial angular velocity $\dot{q}(0)=[1.0\;,1.0]$ are designed respectively. Considering that the spraying material is in the start-up phase in the first 1 s, the delay time of the extended ReT is set as $T_{s}=2\;s$, the convergence time of the PreDT convergent SMSur is $T_{sc}=1\;s$, and the convergence time of the PreDT convergent super-twisting controller is $T_{cp}=1\;s$; thus $T_{pre}=T_{sc}+T_{cp}=2\;s=T_{s}$. Numerical simulations are carried out based on MATLAB 2018a programming with a simulation duration of 10 s. To make the numerical simulation results more consistent with practical engineering applications, the total simulation time interval [0 s, 10 s] is divided into equal intervals of 0.01 s. The ODE45 solver is adopted in each subinterval, and the output value of each subinterval is used as the initial value for the next subinterval in the calculation. The numerical simulation results are shown in Figure 3, Figure 4 and Figure 5, where ATE denotes the angular TraT error.

The numerical simulation results demonstrate that, with the super-twisting SMCler designed in this paper, the actual output angles of the construction robot at different initial positions can accurately track the extended ReT within the PreDT $T_{pre}=2s$. Moreover, the actual convergence time is less than 2 s, which ensures that the construction robot can stably and accurately track the ReT after 2 s, with the tracking accuracy reaching $3\times 10^{-6}$ rad. This verifies the effectiveness of the control strategy proposed in this paper in achieving high-precision TraT.

In the actual spraying process, the actions of the fluid material such as start-up, flow and shutdown will induce parameter perturbations in the construction robot. Meanwhile, the flow of the fluid material will impose various external environmental disturbances on the manipulator, including frictional forces and external pressure. Nevertheless, under the action of the new proposed control strategy, the TraT error of the construction robot still converges rapidly in an exponential manner. No oscillation phenomenon or singularity problem occurs in the system during the entire control process, which fully demonstrates that the novel algorithm possesses strong robustness against the characteristics of the fluid material and external disturbances. It significantly mitigates the chattering problem of the control torque and effectively avoids the occurrence of singularity problems, thus further verifying the effectiveness and excellent control performance of the proposed algorithm.

### 5.3. Comparative Simulation of Different Controllers

To demonstrate the superiority of the newly proposed PreDT convergence property, comparative simulations are conducted between the PreDT convergent super-twisting SMCler proposed in this paper (Predefined-Time Super-Twisting Sliding Mode Controller, PTSTSMC) and the PreDT SMCler in Ref. [23] (Predefined-Time Sliding Mode Controller, PTSMC). Since the strategy of extended ReT is not adopted in Ref. [23], for the sake of uniformity, the extended ReT strategy is uniformly employed for the ReT, with the controller still adopting the one in Ref. [23], i.e.,

(50) $$u=\left\{\begin{array}{cc} D_{0}(\frac{1-\eta }{t_{\mathrm{pred}}-t}\dot{e}-f(q)-Ksign(s)-\frac{\eta s}{{\left(t_{\mathrm{pred}}-t\right)}^{2}}) & t<t_{\mathrm{pred}}\\ D_{0}(-f(q)-Ksign(s)-K_{1}s-\dot{e}) & t\geq t_{\mathrm{pred}} \end{array}\right.$$

(51) $$f(q)=-{D_{0}}^{-1}(N_{0}\dot{q}+G_{0}),s=\left\{\begin{array}{cc} (t_{\mathrm{pred}}-t)\dot{e}+\eta e & t<t_{\mathrm{pred}}\\ \dot{e}+e & t\geq t_{\mathrm{pred}} \end{array}\right.$$

**Figure 6:**
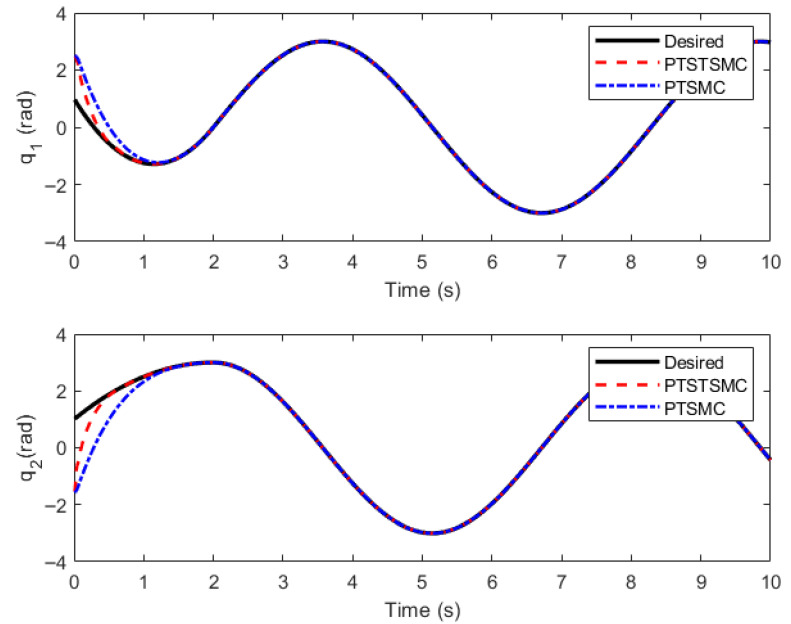
Diagram of angle tracking.

**Figure 7:**
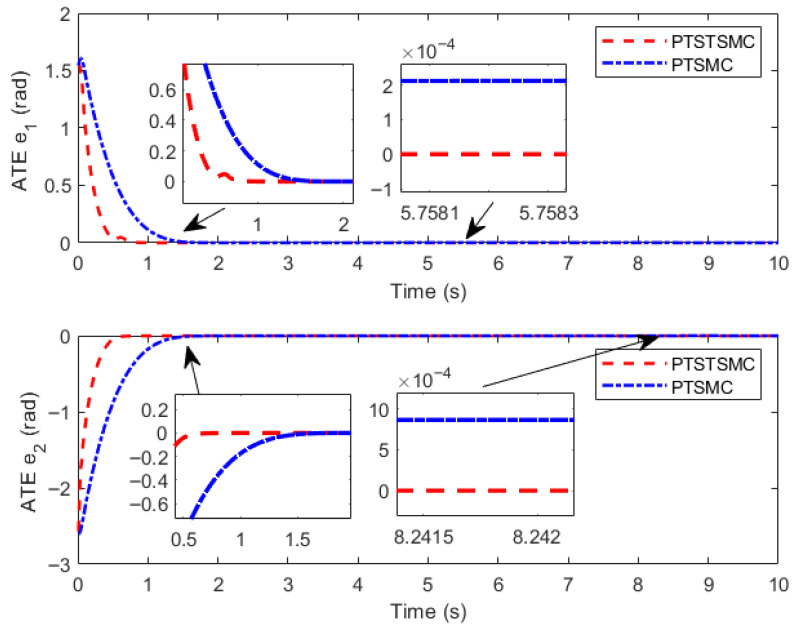
Diagram of angle tracking error.

**Figure 8:**
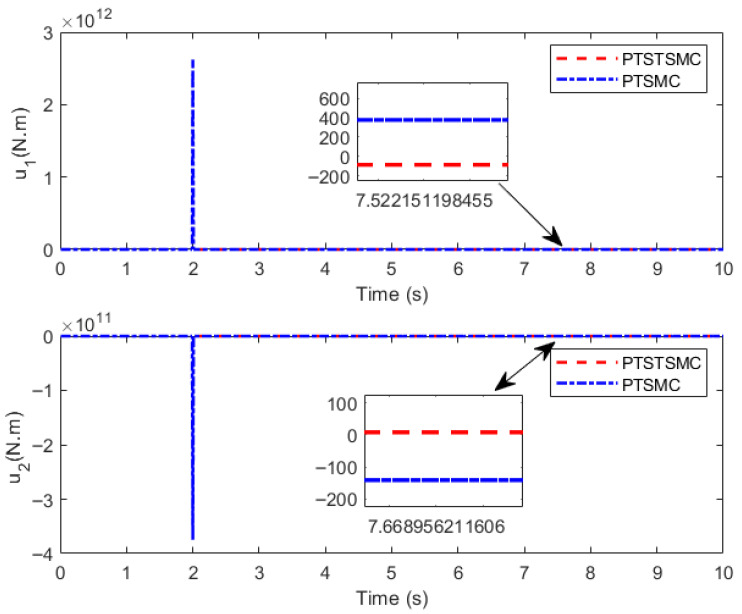
Control input.

Numerical simulations are carried out based on MATLAB, and the results are shown in Figure 6, Figure 7 and Figure 8.

The simulation results indicate that both PreDT convergent controllers can ensure the TraT error of the construction robot converges within the PreDT $T_{pre}=2\;s$, thus guaranteeing the robot’s accurate tracking of the actual ReT. However, simulation results indicate that the PTSTSMC algorithm proposed in this paper exhibits significant performance advantages: its TraT error converges faster, the tracking accuracy is higher, and the required control torque amplitude is smaller. In contrast, the PreDT controller PTSMC presented in Ref. [23] shows an obvious jump phenomenon of tracking error at the extended trajectory connection point t=2 s. Through in-depth analysis, this phenomenon is mainly attributed to the piecewise function structure adopted by the PTSMC controller, which fails to ensure the continuity of the control input at the piecewise points and has a singularity problem. This leads to severe fluctuations in the controller output at the moment of switching, thereby causing a large jump in the controller at t=2 s.

### 5.4. Effectiveness Analysis of RBF Network Compensation

**Figure 9:**
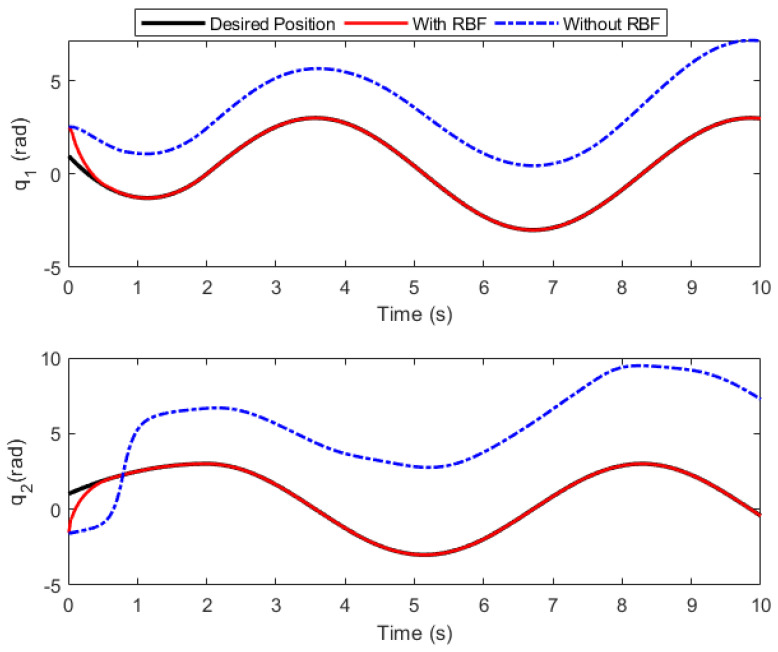
Diagram of angle tracking.

To assess the efficacy of the RBF in controller compensation, comparative simulations are conducted for the controllers with and without the RBF compensator under the same control conditions and controller parameters, and simulation results are presented in Figure 9. From this figure it can be seen that the closed-loop system output fails to converge to the extended ReT in the absence of the RBF compensator, which verifies the effectiveness of the RBF network in resisting external disturbances and improving the TraT accuracy.

**Figure 10:**
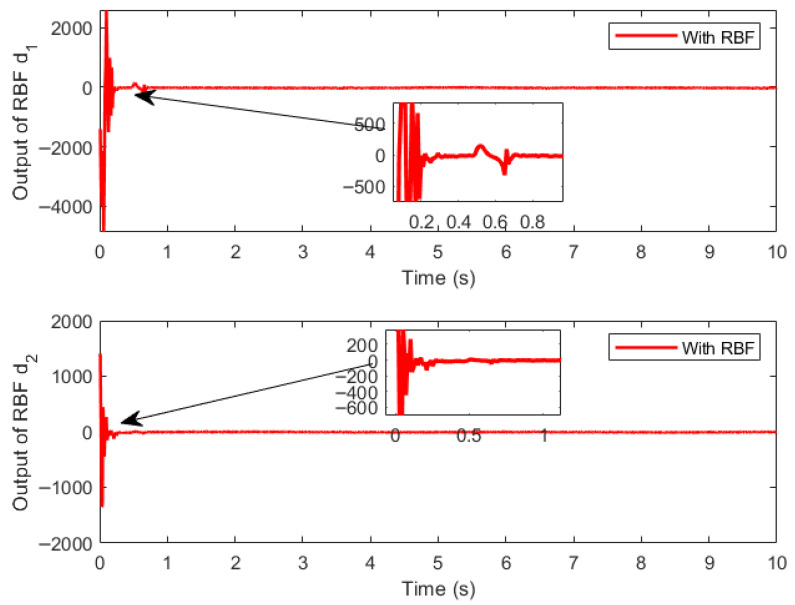
Output of RBF.

**Figure 11:**
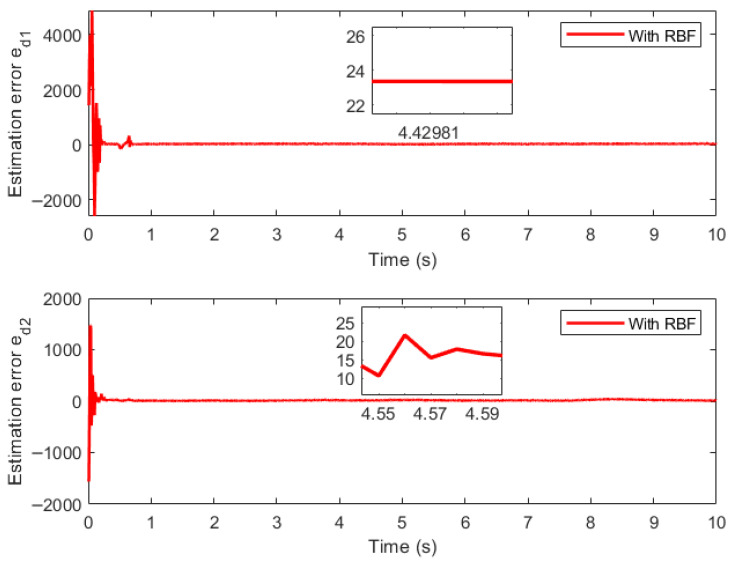
RBF estimation error of disturbance.

**Figure 12:**
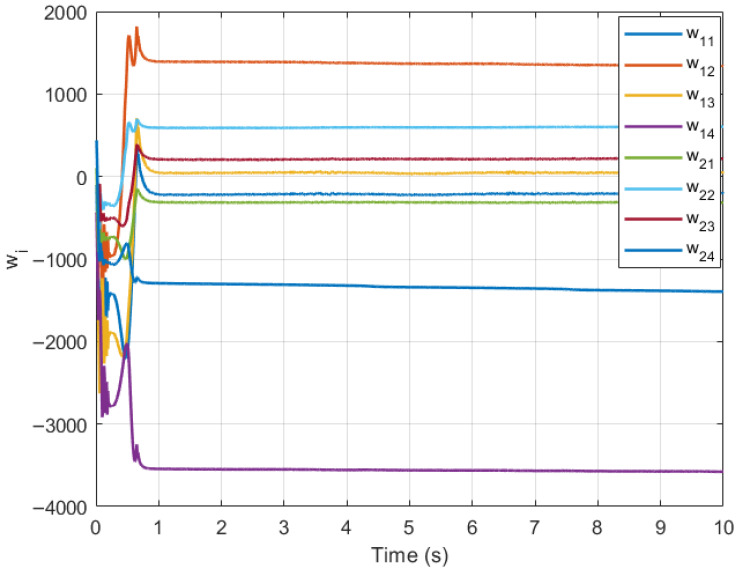
External weight changes in RBF.

Figure 10 shows the output diagram of the RBF network, Figure 11 presents the estimation error diagram of the RBF network for compound disturbance estimation, and Figure 12 depicts the variation trend diagram of the RBF network’s external weights. It is shown that the RBF compensator can effectively estimate the compound external disturbance, and the external weights of the RBF network exhibit the characteristic of fast convergence during the entire control process.

## 6. Conclusions

In practical engineering application scenarios, construction robots are required to achieve high-precision and complete tracking of a given ReT, yet the initial values of TraT errors are typically random. To address this technical challenge, this paper proposes a PreDT convergent super-twisting SMC strategy based on the extended ReT. The specific research contents are as follows:

(1) The influence mechanism of the spraying material flow on the dynamic model of construction robots is explored in depth, and its control characteristics are systematically analyzed. On this basis, an innovative ReT extension strategy and an extended trajectory design algorithm are proposed, and a super-twisting SMCler with PreDT convergence characteristics is designed. This scheme successfully solves the key problem of accurate tracking of the ReT with actual angles for uncertain construction robots in engineering applications. Numerical simulations show that the proposed control strategy not only has effectiveness but also exhibits good universality, and is applicable to various other controllers.
(2) A novel super-twisting SMC theory is proposed, which can ensure the convergence of the sliding surface within the PreDT and greatly enriches the theoretical system of high-order SMC. Based on the designed PreDT nonsingular sliding surface, a PreDT convergent super-twisting SMCler suitable for construction robots is developed. This controller not only effectively solves the singularity problem existing in the traditional PreDT convergent sliding surface but also significantly mitigates the chattering phenomenon in the control process of construction robots, thus improving the control performance.
(3) The RBF neural network is used to approximate the unknown parts in the dynamic system of construction robots, and an adaptive law for the weight vector with convergence characteristics is designed for the RBF neural network. This design enables the RBF neural network to quickly converge to the unknown parts and greatly improve the approximation speed. In addition, the super-twisting SMCler constructed based on the RBF neural network significantly enhances the resistance of the construction robot system to spraying material flow and external disturbances, and effectively improves the robustness of the system.

## Figures and Tables

**Table 1:** Parameters of manipulator.

Parameter	Values	Unit	Parameter	Values	Unit
arm mass	$m_{10}=30,m_{20}=20$	kg	maximum flow velocity	$v_{f\max}=5$	m/s
arm length	$l_{10}=2,l_{20}=1$ $l_{c10}=1,l_{c20}=0.5$	m	start/stop time	$t_{1}=1,t_{2}=9$	s
moment of inertia	$I_{10}=2.93,I_{20}=1.2$	kg.m^2^	density	$\rho_{fluid}=3000\;$	kg/m^3^
gravitational acceleration	g = 9.81	m·s^−2^	pipeline radius	$r_{pp}=0.00757$	m
external disturbance	$\bar{d}=\left[\begin{array}{l} 3\cos(3t)+4\sin(0.9t)\\ -1.5\sin(0.6t)+3\cos(1.5t) \end{array}\right]$	N.m	total pipeline length	$L_{fluid}=l_{10}+l_{20}$	m
ReT position	$x_{r}=[\sin(3t),\cos(3t)]$	-	friction coefficient	$\mu_{0f}=0.3$	-
flow rate	Q=0.017	$m^{3}/s$	coefficient	$k_{\upsilon }=0.013$	-
viscosity	$\mu_{P}=0.3000$	kg/(ms)	angle	$\phi =\frac{\pi }{18}|\sin(3t)|$	rad
moment arm	$r_{fi}=r_{pi}=r_{i_{i}}=l_{ci0}$	m			

## Data Availability

All data are included in this article.

## Abbreviations

ReT	reference trajectory
SMC	sliding mode control
SMSur	sliding mode surface
PreDT	predefined time
RBF	radial basis function
UperB	upper bound
TraT	trajectory tracking
SMCler	sliding mode controller
